# Impact of the institutional model on psychiatric patients in Chile from the 19th to 21st centuries: a scoping review

**DOI:** 10.3389/fpsyt.2023.1114738

**Published:** 2023-09-18

**Authors:** Ennio Vivaldi Macho, Gustavo Gomez Barbieri, Hernán Lechuga, Mauricio Soto-Suazo

**Affiliations:** ^1^Department of Psychiatry, Psychiatric Institute “Dr. José Horwitz Barak”, Santiago, Chile; ^2^Pregrade Department, Universidad Finis Terrae, Santiago, Chile; ^3^Supreme Court Expert, Santiago Chile Children's Surgeon, University of Chile, Santiago, Chile; ^4^Center for Research in Medical Education and Health Sciences, Faculty of Medicine, Finis Terrae University, Santiago, Chile

**Keywords:** mental health, psychiatry, mental health services, Psychiatry/history, mental disorders, Biological therapy/history, Chile

## Abstract

**Introduction:**

Various mental health hospital models have been tested in Chile since its foundation. The institutional model with the Asylum and the Madhouse prevailed during the nineteenth century and much of the twentieth. But is deinstitutionalizing all psychiatric patients the solution?

**Evidence acquisition:**

A PubMed, Epistemonikos, Lilacs, and Google Scholar Scoping Review was carried out in the last 5 years using the PRISMA-P method and the Scoping review search strategy. The MeSH terms (“Psychiatry/history” AND “Chile”) OR (“Mental disorders” AND “therapy”) were used during the search. Finally, papers focused on clinical trial therapy evaluation were excluded, and we emphasized the effects of historical evidence.

**Evidence synthesis:**

We identified 35 primary studies, and we counted the number of articles included in the review that potentially met our inclusion criteria and noted how many studies had been missed by our search. We analyzed 10 primary studies and 10 primary historical resources that were included in this study.

**Conclusion:**

The state must become a guarantor and be responsible for its psychiatric patients and provide professional and humanitarian support to its patients, be it through community psychiatry, day hospitals, devices such as mental health clinics, or psychiatric institutes dedicated to teaching and research. Patients should not be left to the free will of their direct relatives, but rather the state should strengthen the primary care system.

## Highlights

- Historical analysis of the causes, management, and consequences of a mental illness pandemic is crucial.
- The study provides key data on the determinants of health and the organization of variables in the socio-economic system, the organization of health systems, geographical and environmental factors, and genetic factors.
- Limitations: This scoping review uses both scientific literature sources and gray literature in its preparation, We also used historical sources derived from universal literature, which adds a subjective component to the appreciation of the authors of their time when describing the epidemiological phenomenon.
- Final result: We identified 35 primary studies and counted the number of studies included in the review that potentially met our inclusion criteria, noting how many studies had been missed by our search. We analyzed 10 secondary studies and 10 primary historical resources, which were included in the scope of this study. It would be advisable to create a Medical Institute Neuromodulation Center Assessment that studies and controls in a coordinated manner, especially in countries with lower per capita income, such as third-world countries where there is a greater risk of the appearance of mental health conditions.

## Evidence acquisition

**Figure 1:**
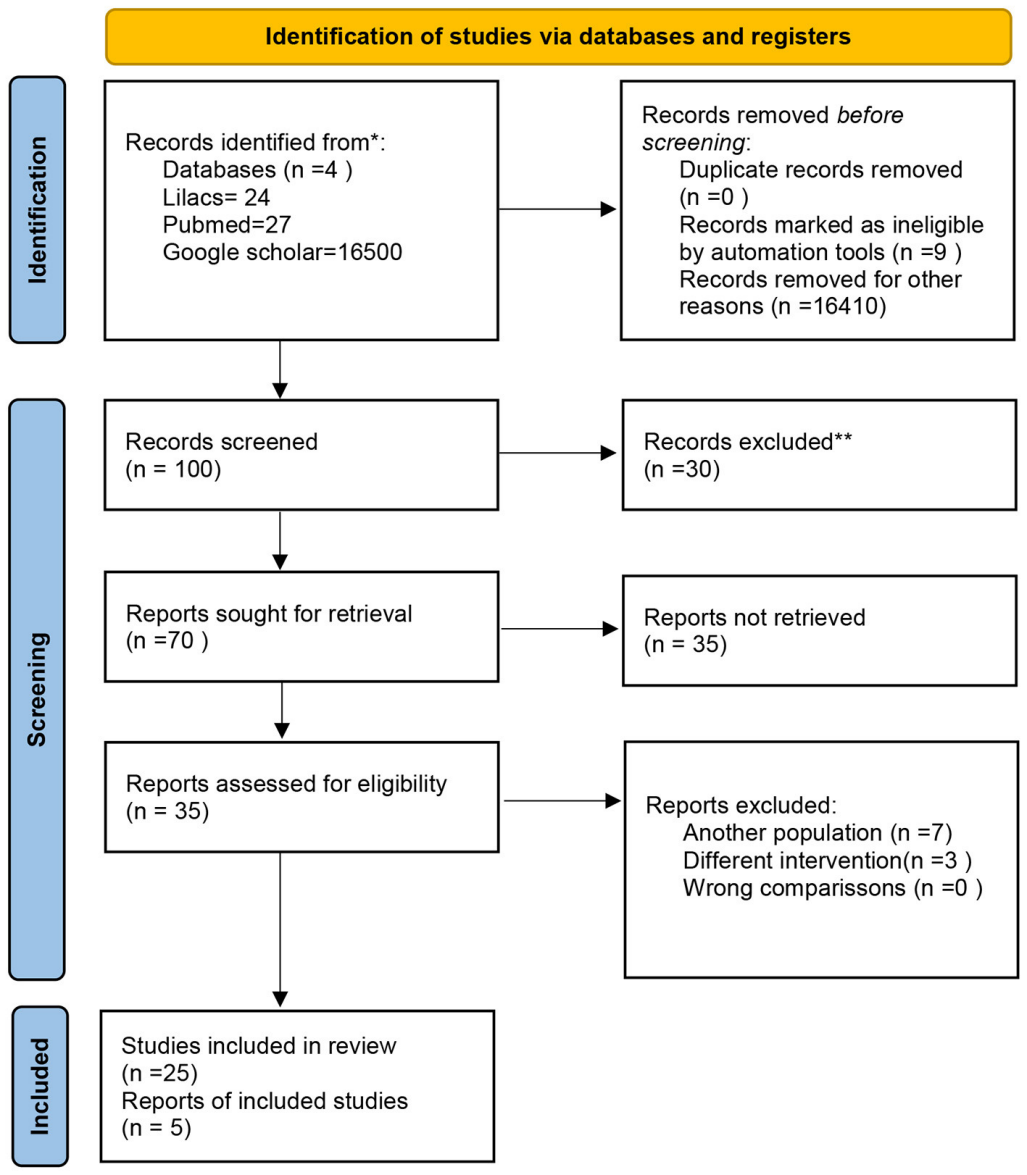
Research question based on the scoping review methodology. *Consider, if feasible to do so, reporting the number of records identified from each database or register searched (rather than the total number across all databases/registers). **If automation tools were used, indicate how many records were excluded by a human and how many were excluded by automation ^=^(“Psychiatry/history” AND “Chile”) OR (“Mental disorders” AND “therapy”). ^=^(“Psychiatry/history” AND “Chile”) ithumcapition uh/.

This literature review aims to illustrate, compare, and discuss the mechanisms through which psychiatry affects social development and health systems in the long term. To achieve this goal, we adopted the Preferred Reporting Items for Systematic Reviews and Meta-Analyses [PRISMA] methodology (1, 2). First, we defined a list of keywords that express the main aspects of the concepts of (“Psychiatry/history” AND “Chile”) OR (“Mental disorders” AND “therapy”) (“MENTAL ILLNESS” AND “INSTITUTIONALIZATION”) OR (“ASYLUM” AND “COMMUNITY PSYCHIATRIC”). Only articles in languages other than English, Spanish, and Italian were excluded. Once the information was obtained, two independent observers, whose names were concealed, carried out a critical analysis of the referenced documents. The original documentary archives of the National Archives of Chile and Italy were consulted to obtain primary sources for a more in-depth analysis (You can see box and Figure 1 for more considerations).

## Methods

We conducted a scoping review of the literature on the impact of the astylar model in Chile, following the Preferred Reporting Items for Systematic review and Meta-Analysis extension for Scoping Reviews (PRISMA -ScR) guidelines (3). A pre-search scoping protocol was designed based on the approach suggested by Arksey and O'Malley. The PRISMA-ScR checklist and protocol are available in the Supplementary material (4–8).

### Eligibility criteria

Current recommendations are as follows ((3, 5)): the inclusion criteria of scoping reviews should be based on the mesh terms that can answer the study questions, the concept to be examined, and the context in which the review takes place. In our review, we applied the following inclusion criteria.

- Primary Historical Studies: Three main questions were structured. What was the impact of asylum model care on the history of Chile? What were the most important measures taken to deal with them and what were the benefits? Are there any drawbacks to the asylum model vs. the community health model? We took into account the terms of the mesh: “Mortality,” “Fatality,” “Psychiatric Hospital,” and “House of madness,” focusing on the implementation of hospital centers and the measures they took to solve the problems of overcrowding, mortality, and lethality within psychiatric establishments. In the second question, the terms used were: “Psychiatry/history” “Chile,” “Mental disorders,” and “therapy,” emphasizing how these measures were implemented in the historical context and finally in the third question: “Authors; Authors; First Chileans; History; first publications psychiatric Chilea; Psychiatry publications Biological therapy/history; elaborating from the verifiable consequences of the texts and the statistical evidence of the impact they had on the control of these conditions (Please review Box 1, 2).
- Secondary historical studies: Autobiographies, historical writings (books and articles/letters) and comments, tributes, photographic archives, legal codes of the time and their translations, study articles, and academic programs following the scoping review methodology with the questions raised were considered among this group.

Box 1 **Research question based on the scoping review methodology.**

**Table:** 

**Question number**	**Research question**	**Quotes that were used answer these questions**	**Data elements**
1	What was the impact of the care of the asylum model in the history of Chile?	1–10	“Mortality” “Lethality” “Asylum” “House of madness”
2	What were the most important measures taken to address them?	11–20	“Psychiatry/history” “Chili” “Mental disorders” “Therapy”
3	What advantages and disadvantages can we find between the asylum and community health models?	21–35	Authors; Authors; First Chileans; History; History; First Chilean psychiatric publications; Psychiatric publications Biological therapy/history

Box 2 **Comparison of models of psychiatric asylum institutions in Chile.**

**Table:** 

**Name article**	**Article type**	**Característica de la institución Asilar**	**References**
The psychiatric ideas of Jose Ramón Elguero, M.D., a physician at the “Casa de Locos” [(the House of Mads) in Santiago, 1862 (author's transl)]	Historical article. A descriptive study of the History of Medicine. Secondary historical source. Assylar model.	Modelo Asilar this work describes the origin of the different locations that Casa de Orates (Madhouse) has occupied Chile. The locations of this institution in the Yungay and Chimba neighborhoods area are specially analyzed. Moreover, the sad and poorly known incident involving the national Madhouse of Olivos and Providencia is narrated.	(9)
Ignacio Matte Blanco, MD, and the development of psychiatry teaching to medical students	Biographical historical article. A descriptive study of the History of Medicine. Secondary historical source. Psychoanalytic Model- Assylar model.	The father of psychoanalysis in Chili and the medical psychiatry education method, were exposed not only in Chile but to the Pan-American Health Organization. He advocated decreasing the time spent in lectures, and increase clinical practice and group dynamic experiences centered on the students. He insisted that teaching had to be focused on issues useful for general physicians and non-psychiatric specialists, as well as in the need to extend the psychosocial curriculum to the internship.	(10)
The contributions of Professor Armando Roa to Chilean psychiatry	Biographical historical article. A descriptive study of the History of Medicine. Secondary historical source. The transitional model between assylay and communitary psychiatry.	Professor Armando Roa had an original conception of the clinical-phenomenological method of investigation in clinical psychiatry, differing from that applied in European clinical studies. Considering that the psychiatrist must rely on clinical facts, the generic features of symptoms must be studied and the way a symptom is lived must be specified. In this way, Professor Roa made five descriptions of normal and pathological anxiety, obsessions, phobias, autism, larvate psychical forms of epilepsy, primitive perception of reference, psychopathy, and anorexia nervosa. He created the concepts of communicative and indicative language, destroyed thinking and unwillingness in schizophrenics, notifying language in neurotics and awareness and the notion of disease. He also made a new classification of alcoholics.	(11)
José Juan Bruner (1825–1899): una estrella fugaz en la historia de la psiquiatría chilena	Biographical historical article. A descriptive study of the History of Medicine. Secondary historical source. Assylar model.	The contribution of Dr. Bruner to psychology and psychiatry is largely unknown. This is a summary of the ideas proposed in his “Medical-Psychological Monograph” from 1857, that which written after a case of a possibly possessed woman from Santiago. In this work Dr. Bruner discards the spirit-brain duality, proposes a functional morphology of the brain, recognizes the importance of remote history-taking when interviewing patients and, proposes a theory for self-formation and the risks of self-fragmentation. H	(11)
Approaches for an Archeology of Mental Health in Chili. Spatial and Material Background of the House of Orates, in its first 50 years	Biographical historical article. Descriptive study of the History of Medicine. Archeology. Secondary historical source. Assylar model. House of madness.	From this review, it is realized how the material changes in total institutions, allow us demonstrating the evolution in the development of the discourses around health and psychiatry and its relationship with the socio-historical context of industrial capitalism, linked to the principles of production and understanding of the person based on their usefulness in the productive system, as workers. The first 5-year period of the Casa de Orates offers an overview to understand the dynamics of social and political transformation under the wing of hygiene and the development of industrial society, but always from the construction of the category of subalterns, which is also structured through starting from specific subcategories such as gender and social class, a relevant aspect to understand the internal dynamics of the use of resources and space of those who inhabited that place.	(12)
Historia de la psiquiatría: Casas de locos. Lei sobre la materia.	Legal history article. Primary Historical article	This law defined the indications for hospitalization derived of law 466 of the civil code to institutionalize patients alienated, under medical protection, which could be withdrawn by the guardian who could be a relative or acquaintance that it could be taken care of by defining the place where he would live at the time of hospital discharge. The administration of the Casa de Orates de Santiago will be at charge of a Board of Directors, composed of five members. It also defines the admission and discharge criteria hospitals with assisted discharge in the event that the family member I would like to withdraw your patient.	(13)
History of Chilean Psychiatry	Biographical historical article. Descriptive study of the History of Medicine. Secondary historical source	From (Demonic Psychiatry; Moralist Psychiatry) Franz Joseph Gall (1758–1828) initiates a kind of Brain Psychiatry, father of Phrenology, which relates the shape of the brain and skull to the functions of the mind. of mental illnesses appears with Emil Kraepelin (1856–1926). With Sigmund Freud (1856–1939). Chilean Manuel Antonio Carmona, who initiates the participation of Chilean psychiatry in the historical scenario (1857). E rnst KretErnster (1888–1964). A We owe Karl Jaspers (1883–1969) the introduction of the phenomenological clinical method. Despite the fact that the Casa de Orates was founded in 1852, a doctor named Manuel Antonio Carmona appeared only in 1857, marking the beginning of psychiatry in our country.	Historia de la Psiquiatría chilena. Clase realizada para el Departamento de Psiquiatría de la Facultad de Medicina del Área Occidente de la Universidad de Chile. Texto elaborado en Abril de (14–17) 995.

Anticipating a low number of studies, we kept our review as inclusive as possible; therefore, we have included any type of study design, including qualitative analyses, ethnographies, case studies, and observational and analytical studies.

### Information sources

Our research included standard databases as well as other sources of information. The standard databases included PubMed, Epistemonikos, Lilacs, Scopus, and Google Shopping Review. A Scoping Review has been conducted over the past 70 years. We also search Chilean Memory, the National Archives, the Library of the Psychiatric Institute with unpublished books and documents, and Sky. Finally, we established direct contact with local mental health stakeholders, including policymakers, clinicians, and service users via email.

### Search strategy

To make our search as complete as possible, we include terms with no language or time restrictions. We use terms like (“Psychiatry/history” AND “Chile”) OR (“Mental disorders” AND “Therapy”); (“Psychiatry/history” AND “Chile”) OR (“Mental disorders” AND “Mortality”); (“Madness” AND “Chile”) OR (“Mental disorders” AND “Therapy”) (Figure 1).

### Selection process

Two assessors independently reviewed the titles and abstracts and compared them to the inclusion criteria. Disagreements were settled by consensus or by a discussion with a third reviewer if no consensus was reached.

### Data extraction and management

An extract of data was developed from the Implementation Studies Checklist for Reporting Standards (18) and the framework developed by Proctor et al. (19). Accordingly, for each included study we extracted general characteristics, such as study type, country, and participants, as well as implementation methods and outcomes, such as acceptability, feasibility, fidelity measures, effectiveness, profitability, and sustainability. A detailed taxonomy is included in the Supplementary material. The data were extracted by one team member and verified by another writer (20, 21).

## Body of evidence

(I) What was the impact of the care of the asylum model in the history of Chile?

Origins of the Great Integration Model: The National Asylum.

### The Jesuit pot shop

In colonial times, on 10 January 1803, the Hospicio de Santiago was founded on a farm located between La Cañada and Zanjón de la Aguada. The place was known as the Ollería de Los Jesuitas, named as such because, during the late sixteenth century, the religious order had installed a large factory making pots, adobes, hostesses, etc. Currently, it corresponds to Portugal Street. It was a facility for the treatment of the so-called “morally ill” people; there were 200 beds, and it was attended twice a week by a doctor. There were two departments, one for men and one for women, and a school that had 300 students. This establishment was managed by the Sisters of Charity. Until then, the people in the city struggling with mental health remained locked up in their homes, in jails, or wandering from town to town, although the richest were sent to Lima. In 1838, a semi-demolished pavilion had been enabled to house some patients of the San Juan de Dios Hospital. It is presumed that the claims of the doctors of San Juan de Dios of the presence of these psychopaths have influenced the foundation of a House for the Insane (22).

### The house of madness “Nuestra Señora de Los Ángeles”

In 1852, during the government of Manuel Montt, the House of Madness “Nuestra Señora de Los Ángeles” was inaugurated in Santiago in the Yungay neighborhood. Its construction was proposed by Lieutenant Colonel Francisco Ángel Ramírez, who was sent on a visit to Lima, Peru, where he visited the House of Madness “San Andrés in Lima,” Peru.

In its beginnings, this institution depended on the Municipality of Santiago, and its first administrator was Sótero Calvo. In those years Santiago had 100,000 inhabitants.

Its management was not exempt from problems due to the scarcity of resources and very high demand. Its last administrator in 1854, the priest Don Juan Ugarte, resigned from the position of President of the Junta, denouncing “the misery and abandonment of the sick,” “crowded like filthy pigs in a corner of the republic and worse condition than the most infamous of criminals,” thus ended his resignation letter (23).

(II) What were the most important measures taken to deal with them?

Law of Internment of Insane or Law on Matter or “HOUSE OF ORATES (MAISON DES ALIÉNÉS)”

It was the first Mental Health Law in the country, published by the government of the President of Chile, Don Manuel Montt Torres, on 31 July 1856. This law is based on the French Law of 30 June 1838, signed by King Louis-Philippe I of France in Neuilly.

This law defined the indications for hospitalization derived from Law 466 of the Civil Code to institutionalize alienated patients under medical protection, which could be withdrawn by the guardian, who could be a relative or acquaintance who could care for them, defining the place where they would live at the time of hospital discharge.

**Figure 2:**
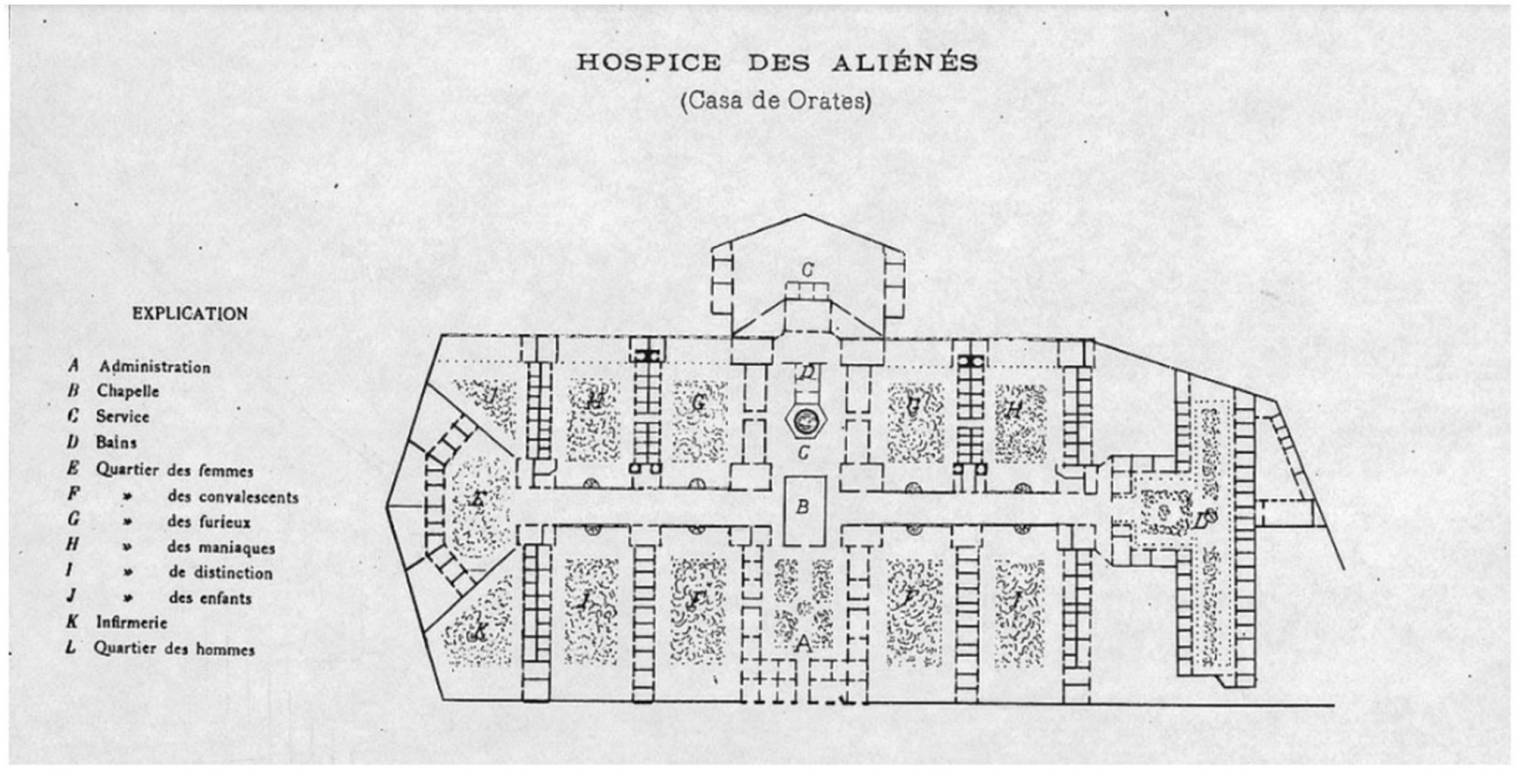
Explanation: A. Administration/B. Chapel/C. Services/D. Bathrooms/E. Women's Sector. F. Convalescent Sector/G. Furious Sector/H Manic Sector. I. Sector of Distinction/J. Children's Sector/K. Nursing/L. Male Sector.

**Figure 3:**
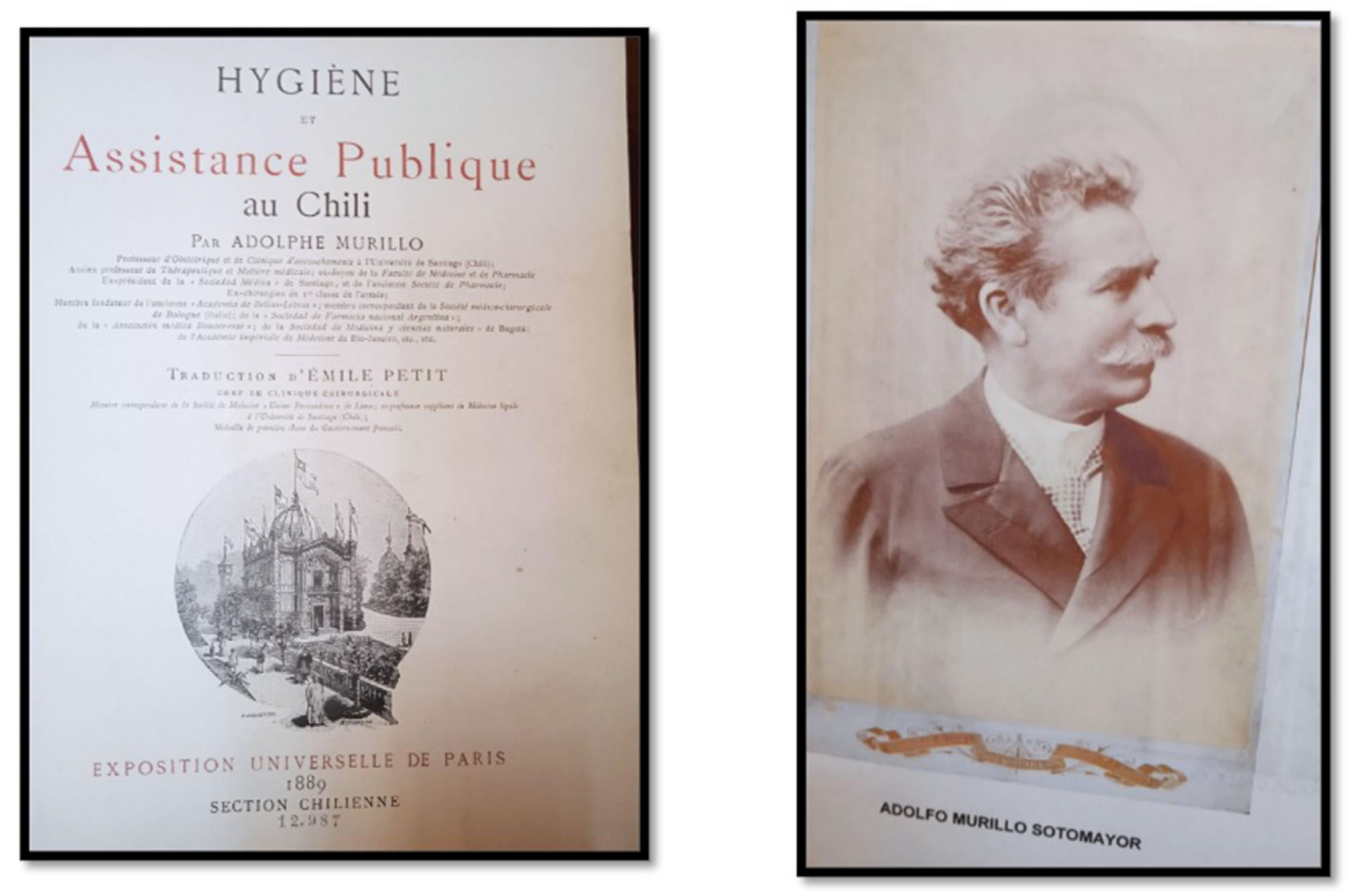
Adolfo Murillo Sotomayor. Portrait. Hygiene and public assistance in Chile. Exposition Universe of Paris, 1889. Time series of hospitalizations for psychiatric causes between 1875 and 1884; the behavior of the curves shows a seasonality every 2.2 years with an exponential increase in hospitalizations around the years 1882 to 1884 that was maintained until the middle of the twentieth century. Its trigger was the War of the Pacific, which began in 1879. Hospitalized foreigners represented 6.9% of all hospitalizations.

The administration of the Casa de Orates de Santiago was in charge of a Board of Directors, made up of five members, appointed by the President of the Republic: a president who appoints people to positions of trust on the committee, a deputy secretary who maintains the minute books and writes them so that they are the faithful expression of what was dealt with and the board agrees; a delegate who oversees the conduct of nursing home officials, a treasurer who manages the nursing home's accounts and finances, and a medical clinical director who directs health officials and is in charge of treating patients (13, 24, 25). For the dawn of psiachiatry in Chile (see Figures 1—3).

### The house of Orates of Santiago

As a consequence of the insufficient capacity to attend to the widespread needs of patients, a four-block plot of land was purchased from the Archbishopric, and the architect Fermín Vivaceta was commissioned to build it in 1854. Finally, it should be noted that the inauguration of La Casa de Orates took place only on 12 September 1858 and that its construction was in the charge of the prominent Chilean architect Don Fermín Vivaceta Rupio: “it was agreed to buy for $8,000 a plot of 4 blocks of land owned by the Archbishopric in the Recoleta neighborhood” (dated 3 November 1854) (24, 26).

### Doctor Juan Marconi Tassara: origins of community psychiatry

**Figure 4:**
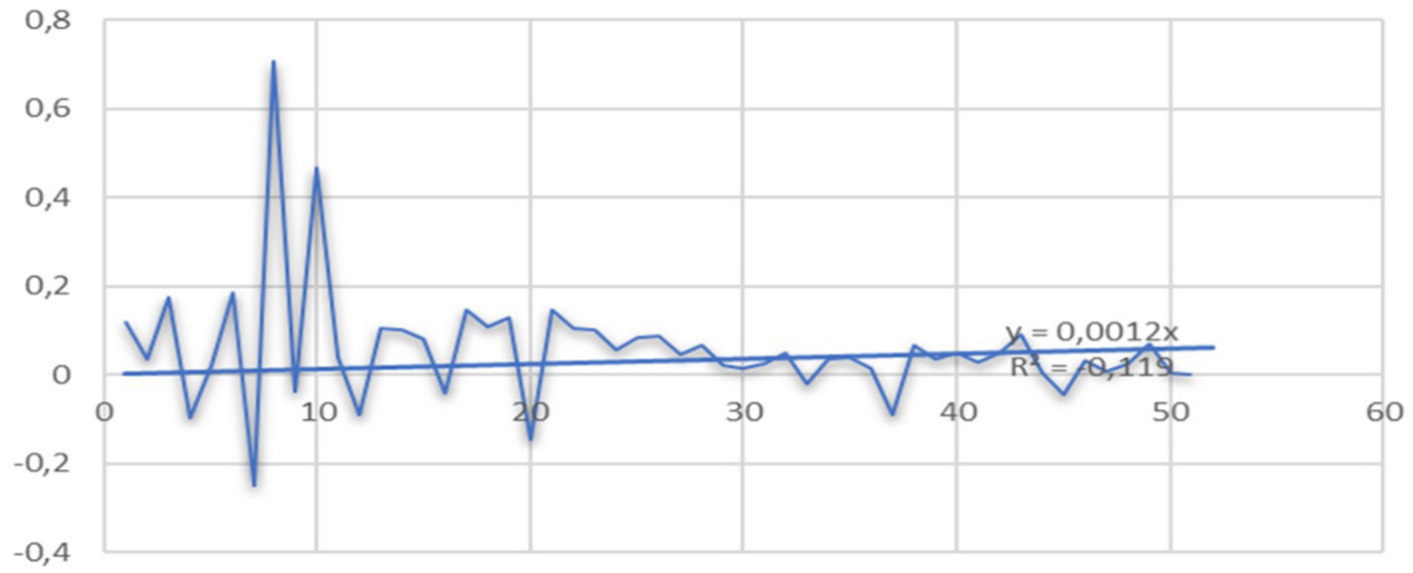
Ratio of psychiatric hospitalizations in the House of Orates in Chile years 1852–1926.

**Figure 5:**
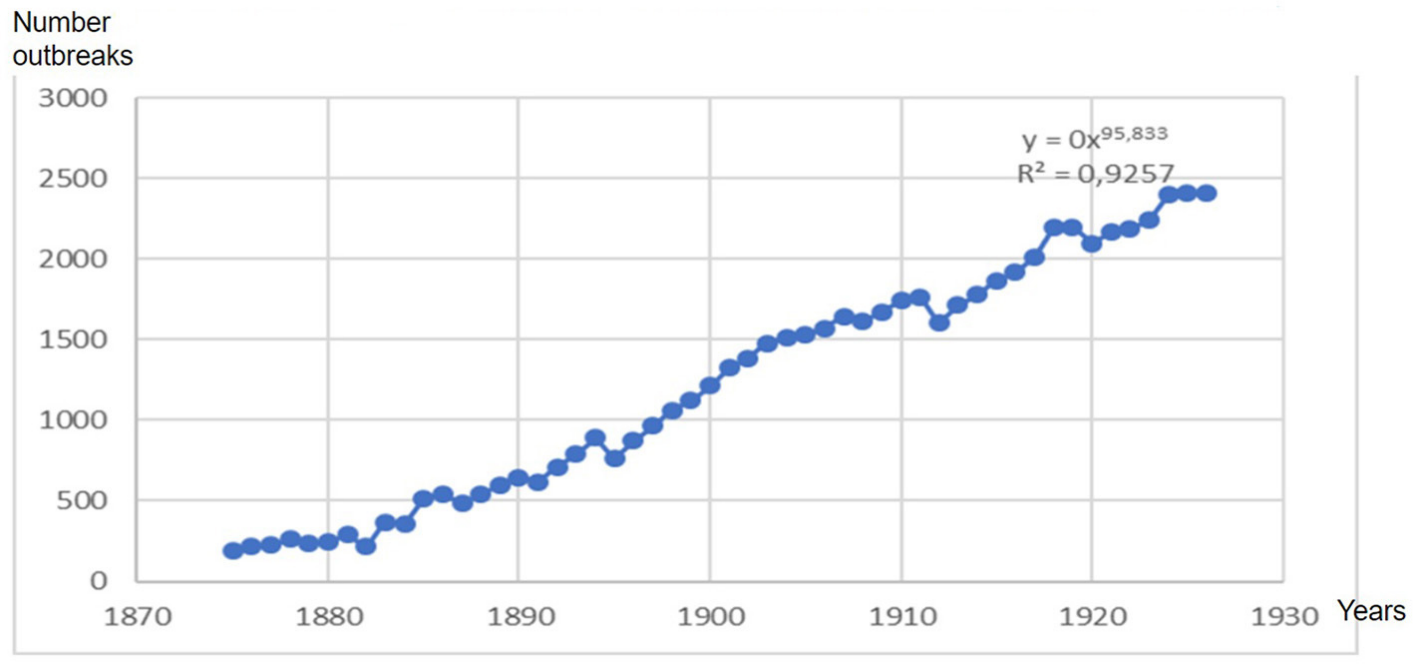
The exponential increase in hospitalizations for mental illness saw a sustained increase between the years 1874 and 1910, presenting a rate of 95.83, which tended toward saturation derived from the fixed capacity of beds, which reached 2,502 beds in 1926.

At the national level, within medicine and psychiatry, the figure of Dr. Juan Marconi Tassara stands out. During his life, he showed respect for those with mental health conditions and their families, and between 1968 and 1973 he developed an intervention model that included participation by the community in important health problems such as alcoholism, neuroses, and sensory deprivation that, according to research, benefited the population. Among the professionals with whom Dr. Juan Marconi worked is Dr. Ignacio Matte Blanco, who was the founder of Chilean psychoanalysis and of whom he was a colleague at the National Asylum. Then he joined the group of Dr. José Horwitz, Head of Service at the Psychiatric Hospital, where he learned in depth about the Asylum model with its poverty, overcrowding, and professional abandonment due to the scarcity of human and material resources and, in Marconi's opinion, the lack of respect for the human rights of the chronically mentally ill, “the leprosy bears of the twentieth century” (27). (See Figures 4, 5) for the increased of hospitalizations and outbreaks increased exponential form.

List of psychiatric hospitalizations in the Casa de Orates in Chile, years 1852–1926.

### Isaac and Joseph Horwitz Barack

Children of a young couple who emigrated from present-day Moldova to America because of the pogrom, Isaac and Joseph Horwitz Barack studied at the National Institute and later at the Faculty of Medicine at the University of Chile. Interested in psychiatry, they were hired at the Asylum, after a neurological stay with Dr. Hugo Lea Plaza, in the old San Juan de Dios Hospital. Dedicated to the Asylum, they—as disciples of Professor Oscar Fontecilla—participated in the teaching and public and private practice of neuropsychiatry (22).

Of the works of Dr. Isaac Horwitz, two writings in collaboration with Dr. G Vila stand out, one dedicated to the “moral madmen” (the current antisocials) where he postulates the need for treatment in centers other than the Asylum, with abortion therapy and psychotherapy, and the publication that talks about the protection and safety of alienists at work (22). Now, psychoanalytical psychotherapy is being applied widely, and other tendencies and the psychosomátic conception of the “enfermedades” are on the rise. This consisted of the lifting of repression, which means as we say that the impulses that were kept in the subconscious being admitted to the conscious. Fundamental regulation of analysis is what Freud named the Freedom Association. It consists of flowing narration for part of the patient of all that passes in the mind without conscious selection or elimination (28).

The patient lies on a sofa and the psychoanalyst behind his head. Sessions last from 45 min to 1 h, four to six times a week. With this method, feelings, impulses, fantasies, and ideas that have so far been unconscious will be known (28).

As for Dr. José Horwitz, he worked at the Quinta Buin Hospital and Clinic and was an international consultant for the World Health Organization (WHO). From these positions, he promoted special care for the chronically ill, renewing, despite the economic limitations, workshops for various trades of what at that time was called “labor therapy” (22). All these interventions were delayed and destroyed by the military government, and only the first PHC centers, with very limited medications for the treatment of alcohol dependence in Psychiatric Health Care (PHC) centers and the availability of basic psychoactive drugs (chlordiazepoxide, chlorpromazine, haloperidol, amitriptyline, and imipramine), were preserved.

Access the interactive link of the video History of Psychiatry in Chile using the following:

Link 1: https://www.psiquiatrico.cl/index.php/289-dia-del-patrimonio-2021 (29)

Link 2: https://drive.google.com/file/d/1Ag6UaaiUDZC5aFC_4wr83Nf-4PbkoSh8/view?usp=sharing

What is the law on the recognition and protection of the rights of people in mental health care, what is its objective, and what are its implications?

The purpose of this law is to recognize and protect the fundamental rights of people struggling with mental illness or mental or intellectual disabilities and their right to personal liberty, physical and mental integrity, healthcare, and social and labor inclusion.

Of the 20 million inhabitants in Chile, the prevalence of schizophrenia is 1%, of which 16.8% (or 34,760 patients) between 2005 and 2018 required hospitalization, making the building of 12 discharge centers necessary. Complexes such as the Horwitz psychiatric hospital, with its capacity of 2,502 patients, were at an all-time high.

- Epidemiology and statistics of mental health in Chile and the world

Health is a state of complete physical, mental, and social wellbeing and not simply the absence of disease, Constitution of the World Health Organization, Geneva, 1948.

There is a considerable variety of mental disorders, which have different manifestations. These disorders are characterized by combining alterations in thought, perception, emotions, behavior, and relationships with other people.

Mental health is a state of wellbeing in which the person develops their capacities and can face the normal stresses of life, work productively, and contribute to their community. In this positive sense, mental health is the foundation of individual wellbeing and the effective functioning of the community. Mental health and wellbeing are fundamental to our collective and individual ability to think, express feelings, interact with others, earn a living, and enjoy life. On this basis, the promotion, protection, and restoration of mental health can be considered vital concerns of individuals, communities, and societies around the world.

Yet mental health remains a neglected part of global efforts to improve this problem. People with mental health problems experience widespread human rights violations, discrimination, and stigma; this includes more than 80% of people who suffer from mental disorders.

In Chile there are no current mental health studies focused on health personnel; however, Chile is 1 of the top 10 countries in terms of suicide rates within Latin America. The specific suicide rates for the period 2000 to 2017 were 8.5, 5.4, and 14.7 per 100,000 in the groups 10–24, 10–19, and 20–24 years, respectively. This is closely correlated with the human development index since the Aisén, Los Lagos, Magallanes, and Los Ríos regions scored low on the regional inequality index, showing the highest suicide mortality rates. The highest risk of suicide was estimated in men (RR = 3.5), young people (RR = 2.7), and in the Aisén region (RR = 2.0). The national average suicide rate in the 10–24 age group remained at 8.5 per 100,000 in the periods 2000–2008 and 2009–2017. The greatest geographic inequality was found in men between the ages of 20 and 24 in the period 2000–2008.

The challenges posed by the new Mental Health Law are diverse. Among the most important challenges, we find the small number of hospital establishments, professionals, and multidisciplinary teams dedicated to the development and treatment of psychiatric patients.

One of the main points and consequences of the new Mental Health Law is that by deinstitutionalizing the treatment of psychiatric patients and handing them over to their families, those lacking the means and financial support to carry out their treatments will be forced to join the society and cultural environment of the patient; a professional is brought to respond to the needs of the biopsychosocial patient, communicating with the family group and the medical team, and providing tools for the diagnostic management and treatment of the psychiatric patient.

- Impact of the Law and possible solutions

The Mental Health Law, more than a law, is a manifesto or a letter of principles to guarantee the rights and duties of psychiatric patients, but it leaves open an infinite number of problems that have no solution and that do not manifest and explain how they will make changes.

The current law questions the responsibility of the State, placing the responsibility on them to provide coverage and care for psychiatric patients in highly complex establishments and explaining this to their relatives and their free will, which constitutes a delay in public policies toward the pre-asylum era as described by Michel Foucault in his History of Madness in classical times where mentally ill people were shipped off and discarded to their fate in their towns and where they were dragged by the so-called ship of fools where they were reprimanded in other towns only to, due to their strange customs and habits, be again rejected and taken by the sea and the waves to their perdition. However, with the Renaissance began what is now called the era of the Great Confinement where mentally ill people were institutionalized to remove the insane from the sight of the neurotypical people, thus applying Cartesian-type rationalism, to make “unreason” disappear from our soil however it took root (30).

**Figure 6:**
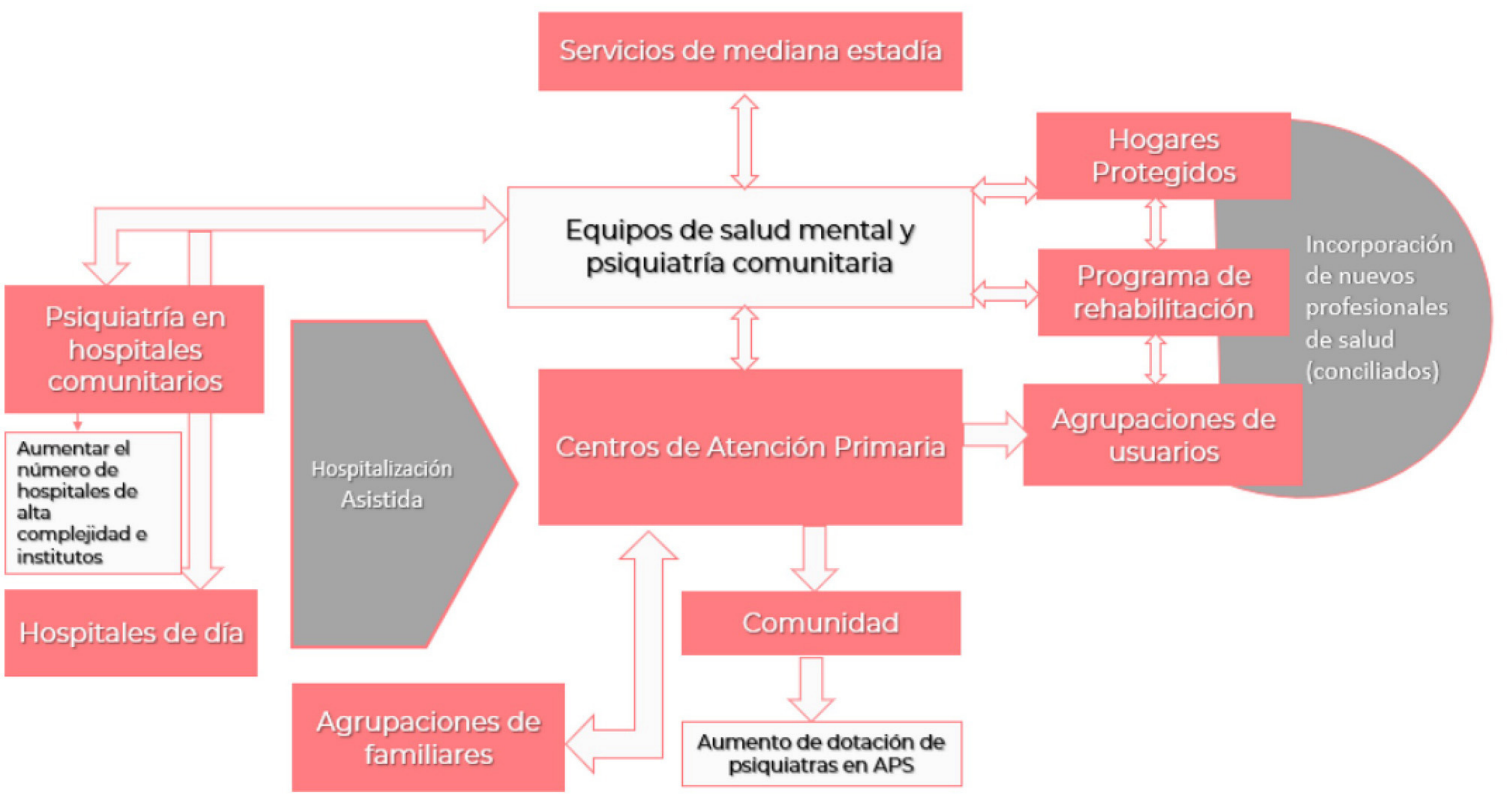
Mental health system 2020–2050.

The aim of deinstitutionalization is to limit the role of psychiatric hospitals, incorporating acute hospital beds into the general hospital and replacing them with community supported living solutions for people experiencing severe mental illness (31). In parallel, an efficient network of community-based mental health services is needed. In the particular case of Chile, there is a deficit of short- and long-stay beds, which is reflected in the significant attention to emergency beds in the central Emergency Room in Chile (Posta Central-Hospital de Urgencia y Asistencia Pública), which does not have the training or capacity to assist these patients. These patients are highly complex and understood to be of a high risk of suicide, self-mutilation, harm to other parties, mutism, etc.; it is necessary to increase the provision of short-stay beds and improve care in the community, strengthening its link with the associations of patients and the Primary Health Attention and Mental Health Center network of relatives, creating a psychiatric emergency hospital as is the case of the Italian hospital in Buenos Aires, which has a triage and ambulance device for containment and rapid hospitalization and integration of short-stay low-to-moderate risk patients, which consist in two weeks to hospitalization with resolution and accompanied by psycotherapist and logotherapist (32). View Figure 6.

### Body of evidence

Box 3 comparison of models of psychiatric asylum institutions in Chile.

Box 3 **Comparison between the different models of psychiatric hospitals in Chile and the world.**

**Table:** 

**Psichiatry hospitalary model**	**Origin country**	**Model type**	**Characteristics**	**PIB destinado a salud mental**	**References**
Mental Health Model with community mental health services in the process of connecting with the Hospital System.	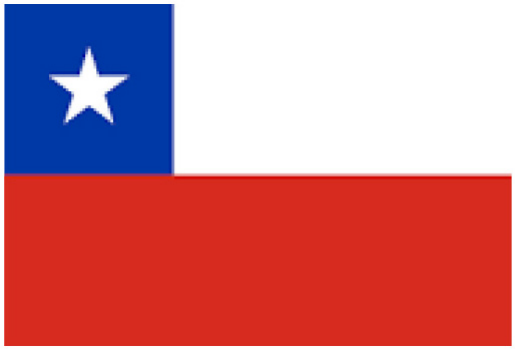 Chile (1852–2022)	Mixed (Community in process of deinstitutionalization since 1955)	The development of the community care model allows available care resources, whether public or private, to be applied to mental health disorders and psychiatric diseases of the population of a certain geographical area in a coordinated and integrated, giving priority to community and participatory strategies, according to the realities and needs of each gender and sociocultural group. According to this model, the axis of organizational support is the community mental health and psychiatry team. It consists of Rural Posts of 1,162 Offices APS 596 Community Centers Mental Health 38 Psychiatry Clinics 57 Day Hospitals 40 Short Stay Psych. General Hospital 19 Psychiatric Hospitals (1) 5 Forensic Psychiatry Services 3 Day Centers 25 Protected Homes 85 Protected Residences 21 Private Psychiatric Clinics 31	2.14% of Total GDP (33.3% hospital network and 64% for the rest of the community benefits; Who-Diprece,2007) and with 0.1 psychiatrists per 1,000 inhabitants, equivalent to 1 psychiatrist per 10,000 inhabitants or 100 schizophrenic patients, of whom between 19 and 35 patients will require hospitalization per year by medical psychiatric personnel.	(33–35)
The institutionalized hospital mental health system integrated into the community network-called Geographic Sectorization.	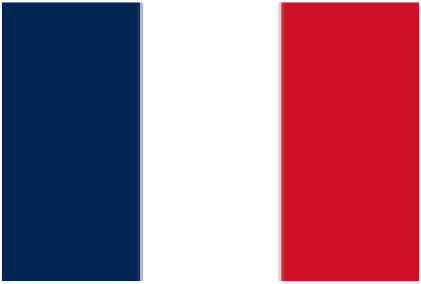 Francia	Integrated hospitalization model Geographic sectorization	In France it is organized according to a geographical sectorization policy. An adult psychiatry center covers an area of 95,000–150,000 inhabitants, while a child and youth psychiatry center cover an area of 140,000–210,000 inhabitants (or ~40,000 young people under 16 years of age). It consists of eight hospital complexes distributed around population density in French Institutions—GHU Hospital Sainte Anne Hospital Maison Blanche Hospital Perray Vacluse ASM 13 Hospitals de Saint Maurice Psicologic Medical centers (CPM)	According to the OECD report, in 2021 15% of GDP Francthe e allocates the item to Mental Health (0.23 psychiatrists, 0.49 psychologists and 0.98, nurses per 1,000 inhabitants, that is, 2 psychiatrists, 5 psychologists, and 10 nurses per 10,000 inhabitants).	(29, 36–38)
United Kingdom (UK)	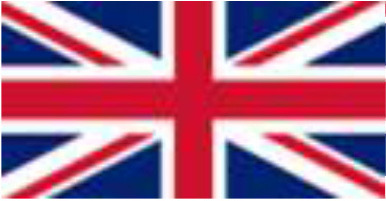 UK	Institutionalized Trans-Community Psychiatric Model	There is a statement, contained in the modernization of mental health services, that health law for England and Wales would change with a view to forcing people at risk with mental health problems problems complying with community treatment, along with efforts to create a new category of mental illness, “danger-severe and severe personality disorder” (38) also announced was a plan to address service deficiencies by establishing new types of community equipment (38), including those that intensive, “assertive outreach” services, with a mandate interact proactively with people at risk. More generally, the identification of the system failure was reflected in actions aimed at correcting the deficiencies through the top-down implementation of standards and frameworks [(28, 31, 39), 45] and clinical guidelines (for example, for care and treatment of people with schizophrenia	According to the OECD report, 2021 10% in of GDP.	(40, 41)
Community health model in Italy	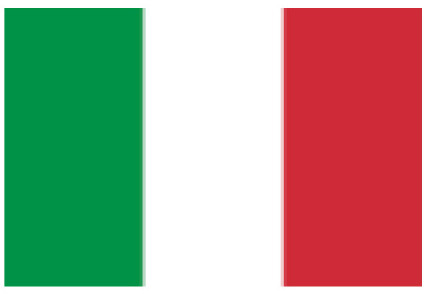 (1978 Trieste-2022 Italia).	Deinstitutionalized community mental health module.	The community psychiatry model in Italy began in Trieste in 1978 in 1978 in Italy, when the application of the psychiatric reform law began, which launched a national process to eliminate psychiatric hospitals “The strength of this system lies in the Mental Health Centers, which operate 24 h a day in four geographical areas of the city, each with eight beds. The Centers provide medical and social care, psychosocial rehabilitation and treatment for acute episodes. For people who need long-term help, apartments (with 55 beds) have been created that accommodate small groups, in a pleasant environment. The Enabling and Residence Service coordinates these apartments, the habilitation and social integration activities, internado en un Hospital Psiquiátrico Judicial,” with workshops and projects throughout the city. By creating job opportunities, it has been possible to guarantee effective integration into the social environment. The number of Compulsory Sanitary Treatments, with an average of 8 per 100,000 inhabitants in the last 10 years, is the lowest recorded in Italy. This then means that no citizen of Trieste is found	According to the OECD report, 2021 3.4% GDP Italy of allocates the item to Mental Health (0.23 psychiatrpsychiatristsychologists and 0.98, nurses per 1,000 inhabitants, that is, two psychiatrists, five psychologists and 10, nurses per 10,000 inhabitants)	(39)

## Discussion

### What advantages and disadvantages can we find between the asylum and community health models?

Since the dawn of the republic, the fundamental unifying concepts of the concept of the nation were imbued by Enlightenment ideas from the Old Continent (42). The awareness of a political, social, and legal order opened the doors to a dialogue between the civil and the institutional spheres, accepting that no regime would solve socio-political problems nor would any effective organization of institutions characterized by a conservative and authoritarian character (43–45).

The drive for autonomy led to self-deliberation in the most varied spheres. The foundation of private schools beyond the domain of religion promoted a radical reform with the very understanding that scientific advances and communication between researchers and progressives would lead to the founding of a liberal republic in the broad sense of the word (45, 46).

From there will be born different variants of what health means beyond the medical field. Social welfare extends beyond health policies to the formation of a social body capable of responding responsibly to its citizens, avoiding unnecessary evils, and acquiring new practices for the management and treatment of patients (47).

Psychiatry will not be the exception, which will deliberately see expansion in therapy beyond confinement itself with a mechanism based on psychotherapy and psychoanalysis, placing the patient in a ubiquitous place of what health and illness mean (48). The legal corpus will recognize the patient as a subject of law, with their guarantees being explicit in their treatment as a person and subject of law, providing them with adequate measures of access to care, treatment, and social reintegration (49, 50).

So far there is nothing new under the sun. Perhaps there will be an order that seeks to reveal some new therapeutic-social-legal treatment that modifies both the psychosocial structure in dealing with the patient. Obstacles have been and will be found in the path of therapeutic care; the lack of adherence to therapy concerning the human person in the legal framework will lead to multiple deficiencies in multiple systems and areas of human development (33).

The asylum model understands the meta-concept of “madness;” this reality “does not have so much to do with the truth and with the world, as with man and with the truth in himself, which he knows how to perceive.” This knowledge leads us down a path of uncertainty (51). If in the Middle Ages the fear was death, in the Modern Age this fear is transformed and transfigured to alter the psychosocial environment. The patient's figuration and knowledge hide a symbolism that does not reflect what is real but attacks and mirrors what society does not want to see of itself. So, do we use the asylum to hide this truth, or, with the adequate institutionalization of the patient, do we fight against what we do not want to see but we know is present all the time? The philosophical experience of “madness” led already in the sixteenth century to the establishment in Germany of the first permanent shelters for those patients who shared a common line of that intricate knowledge that led them to struggle with mental health and did not allow them to assimilate with the social environment (52–54). The so-called workhouses proliferated in Europe in classical times, and through the organized work of psychotherapy, psychoanalysis, and modern medicine they avoided setting the course for what would be community work as therapy for social and labor inclusion. These chronic patients and their activity in craft workshops through labor therapy develop cognitive knowledge that leads them to “normalize their relationship and with the environment” (33). The so-called morally insane, a term modeled by the psychiatrist Isaac Horwitz, are subjects of family medicine since their degree of sociopathy makes them subjects of functional therapy with community effectiveness. That is why, due to their degree of chronicity, they can be treated in centers other than nursing homes. Unfortunately, patients in advanced stages of neuropsychiatric pathology are not subject to a benefit based on a minor outpatient AI, but rather, given the degree of severity and chronicity, they must be constantly monitored in full adherence to their legal rights as people while respecting their autonomy. Special attention should be paid to the depth and chronicity of the patient as well as alterations in their structures of thought, perceptions, emotions, behaviors, and way of relating to the environment, clearly establishing the degree of disability and effectively communicating it to a family member or companion who is responsible for the dialogue with the multidisciplinary team and the one whose provision will contribute to consensual management in the doctor–patient relationship and with adherence to respect for human rights without discrimination or stigma and in the constant search for a degree of improvement that allows the earliest social reinsertion of the patient as a useful and skillful subject for society (30).

From the multidisciplinary point of view, we must point out that unfortunately, the psychiatric patient is not only subject to neuropsychiatric pathologies but simultaneously presents other pathologies of a complex nature. Cardiac, neuromuscular, metabolic, and oncological pathologies generate comorbidities that only aggravate the clinical pictures. All these situations must be adequately controlled and resolved in the field of both psychiatric and hospital emergencies, for which the mother psychiatric unit must be adequately equipped with the specialties capable of absorbing this demand in a self-sufficient way (50, 55).

From the above, we must point out that the institutionalized patient has not only played a negative role of exclusion, but also a positive role in the organization. Its practices and its rules have constituted a domain of experience that sees unity, coherence, and its function (30). It is within this experience that approaches to the organization of the ethical world are achieved, bordering between good and evil, the norms of social integration. It is bodywork, a cultural brief, and a meeting and reorganization place. As if that were not enough, it is the place of academicism and a bridge to the social; it is intervention in real-time with its achievements and consequences and a mirror of society; it is the recognition of man; it is the manifestation of plurality; and it is the greatest sign of dedication and empathy for what the human person should mean (40, 51).

Integration is not capricious or trivial. It is the last resort—but also the first—because who but the right professionals will be capable of making a real-time diagnosis of a pathology hidden in time that seeks to camouflage itself in the nooks and crannies of life? The common ones manifest their presence when the severity is such that the consequences can be disastrous, both for the patient and for their environment. It is then illogical to hand over the great responsibility of a seriously compromised patient, where the responsibility is great and human and material resources scarce; the responsibility belongs to the social corpus, and it is the institution created to absorb this demand that must take charge of public education. It is the governing body's duty and role as a valid interlocutor at the international level by the requirements and opportunities indicated by the moral and legal law since, as the head of the World Health Organization said, “there is no health without mental health (56).” Chile is responsible for the health of its citizens, Chile is the one that must face the disabilities generated by the growth of psychiatric illnesses, Chile will have to train the specialists that we lack to provide care for our population. It is to be reductionist; no, we have to be realistic and provide more support for psychiatry and more support for Chile (39, 41).

## Conclusion

We must generate a synergy between the institutionalized and community health systems, allowing the psychiatric patient to be incorporated into their biopsychosocial environment by providing tools for its strengthening by reducing inequities and inequalities in mental health. This is why the State must become a guarantor responsible for its psychiatric patients and provide professional and humanitarian support to its patients, be it through community psychiatry, day hospitals, devices such as mental health clinics, and psychiatric institutes dedicated to teaching and research without leaving patients to the free will of their direct relatives, cooperating with the families for integration with their families and communities while strengthening both the primary care system and rural posts. They have specialized equipment for various mental pathologies such as depression, suicide, schizophrenia, IA, and anxiety disorders without forgetting to provide new third-level hospital facilities that allow for care and resolution of the most complex pathologies.

## Protocol registers

This review article was no included in the PROSPERO registry because our work does not contemplate intervention in humans or animals, but it is rather a narrative review and analysis of the body of evidence of primary and secondary sources from original historical sources.

## Data availability statement

The original contributions presented in the study are included in the article/Supplementary material, further inquiries can be directed to the corresponding author.

## Author contributions

GG: formal analysis, writing—original draft, writing—proofreading and editing, and visualization. EV: conceptualization, methodology, supervision, and writing—review and edition. HL: methodology and writing—review and edition. MS-S: conceptualization, methodology, supervision, writing—original draft, and writing—review and editing. All authors contributed to the article and approved the submitted version.
